# Microbial Fibrinolytic Enzymes as Anti-Thrombotics: Production, Characterisation and Prodigious Biopharmaceutical Applications

**DOI:** 10.3390/pharmaceutics13111880

**Published:** 2021-11-05

**Authors:** Chhavi Sharma, Alexander Osmolovskiy, Rajni Singh

**Affiliations:** 1 Amity Institute of Microbial Technology, Amity University Uttar Pradesh, Noida 201313, India; sharmachhavi27@gmail.com; 2 Department of Microbiology, Faculty of Biology, Lomonosov Moscow State University, 119991 Moscow, Russia

**Keywords:** microbial fibrinolytic enzymes, enzyme production, enzyme purification, characterisation, structural-functional attributes, thrombolytic potential, biopharmaceutical applications

## Abstract

Cardiac disorders such as acute myocardial infarction, embolism and stroke are primarily attributed to excessive fibrin accumulation in the blood vessels, usually consequential in thrombosis. Numerous methodologies including the use of anti-coagulants, anti-platelet drugs, surgical operations and fibrinolytic enzymes are employed for the dissolution of fibrin clots and hence ameliorate thrombosis. Microbial fibrinolytic enzymes have attracted much more attention in the management of cardiovascular disorders than typical anti-thrombotic strategies because of the undesirable after-effects and high expense of the latter. Fibrinolytic enzymes such as plasminogen activators and plasmin-like proteins hydrolyse thrombi with high efficacy with no significant after-effects and can be cost effectively produced on a large scale with a short generation time. However, the hunt for novel fibrinolytic enzymes necessitates complex purification stages, physiochemical and structural-functional attributes, which provide an insight into their mechanism of action. Besides, strain improvement and molecular technologies such as cloning, overexpression and the construction of genetically modified strains for the enhanced production of fibrinolytic enzymes significantly improve their thrombolytic potential. In addition, the unconventional applicability of some fibrinolytic enzymes paves their way for protein hydrolysis in addition to fibrin/thrombi, blood pressure regulation, anti-microbials, detergent additives for blood stain removal, preventing dental caries, anti-inflammatory and mucolytic expectorant agents. Therefore, this review article encompasses the production, biochemical/structure-function properties, thrombolytic potential and other surplus applications of microbial fibrinolytic enzymes.

## 1. Introduction

Cardiovascular diseases (CVDs) for patients with myocardial infarct, hypertension, hyperlipidaemia, diabetes mellitus, stenocardia or coronary heart disease are dominant causes in the ensuing upsurge of mortality worldwide [1,2,3]. The World Health Organization (WHO) demarcated 17.9 million deaths each year from CVDs, an estimated 31% of deaths globally [4]. Fibrin clots in vivo are formed through activated thrombin (EC 3.4.21.5) and hydrolysed by plasmin (EC 3.4.21.7) as a haemostatic response under standard physiological circumstances to prevent thrombus formation. However, excessive coagulation or irregular fibrin accretion in the blood vessels results in life threatening intravascular clotting, commonly referred to as cardiovascular thrombosis [5]. Anti-thrombotic strategies such as anti-platelet drugs (aspirin and dipyridamole), anti-coagulants (heparin and warfarin) and surgical operations are the keystone of thwarting thrombosis but substantially upsurge the risk of after-effects, specifically haemorrhage [6].

It has been shown that microorganisms can secrete enzymes that demonstrate fibrinolytic activity like plasmin, which makes them more accessible and cheaper producers of such enzymes as compared to their analogues. Advances in studies of these kinds of enzymes open up great opportunities in solving the problem of lysis of preformed blood clots. Therefore, an important stage in such studies is the directed search for new producers that have certain advantages over those already known [7,8].

With the emergence of “Biocatalysis” as an eco-technology, a promising perspective of microbial fibrinolytic enzymes has progressively attracted therapeutic prominence because of their comprehensive biochemical diversity, low expense, scale-up feasibility and easy genetic modification which could directly lyse existing thrombus inside the blood vessel [9,10]. Scientific reports suggest numerous microbial sources of fibrinolytic enzymes such as bacteria (including actinomycetes), filamentous fungi and microalgae [10,11]. A cultivation-dependent methodology is largely employed for screening microbial producers of fibrinolytic enzymes. However, with an aim of enhanced production/high efficacy: recombinants, mutagens and metagenomic libraries are also created as cultivable microbes and are no more than 1% of the entire microbial community [10]. Furthermore, different traditional fermented foods are also testified for producing efficient fibrinolytic enzymes, for example: nattokinase extracted from Japanese natto [12,13] and fibrinolytic enzyme [14] from soyabean fermented Douchi efficiently lysed thrombi in vitro and in vivo.

Conventional and statistical optimisation of nutritional components and physico-chemical parameters—carbon, nitrogen, substrates, minerals, temperature, pH, rate of agitation and inoculum size—are some of the pivotal approaches stated for the fibrinolytic enzyme’s significant fermentative production [15]. Subsequently, with the detection of pH/temperature/organic solvent stable proteolytic fibrinolytic enzymes, the additional unconventional pharmacological and industrial applicability of such enzymes has also emerged. Some of the recent applications suggest their use as functional drugs/food additives to prevent and cure CVDs, anti-microbials, potential detergent additives for blood stain removal and blood pressure regulators along with mucolytic expectorant agents [12]. Thus, microbial fibrinolytic enzymes have not only emerged as potential biocatalysts with tremendous usage possibilities as prodigious fibrinolytic agents, but they exhibit other miscellaneous applications as well.

**Figure 1:**
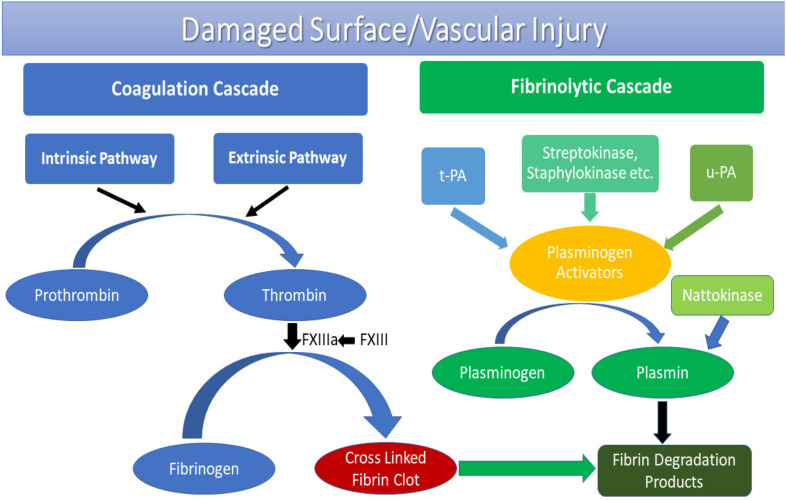
In vivo working mechanism of microbial fibrinolytic enzymes.

Fibrinolytic enzymes (EC 3.4.) of exogenic origin, based on their in vivo working mechanisms are currently distinguished into two categories: plasminogen activators (PAs) and plasmin-like enzymes [16] (Figure 1). The first, being the PAs such as urokinase-type PA (EC 3.4.21.31) and streptokinase (EC 3.4.99.22), convert the inactive precursor of plasmin that is plasminogen into active plasmin and eventually leads to fibrin hydrolyses [17]. The second type of fibrinolytic enzymes are plasmin-like proteins such as nattokinase (EC 3.4.21.62) which directly initiates fibrin hydrolyses and reinstates standard vascular architecture by degrading blood thrombi promptly and completely [17]. Based on the catalytic mechanism, the fibrinolytic enzymes are further categorised as serine protease (for example, Bafibrinase, Terrilitin, *Aspergillus ochraceus* AO-1 protease) [18,19,20], metalloprotease (for example, *Serratia* sp. KG-2-1 metalloprotease) [21] and a mixture of serine metalloprotease (for example, *Streptomyces* protease, *Serratia marcescens* subsp. *sakuensis*) [22,23]. Additionally, intermolecular protein–ligand interactions with an active physiological substrate and activator/inhibitor specification control an enzyme’s cellular/biochemical processes. The structural-functional elucidation of enzymes is henceforth essential to study their mechanism of action and ultimately commercialisation [24].

Regardless of their enormous uses, fibrinolytic enzymes exhibit several inadequacies such as low fibrin specificity, short half-life span, allergic reactions, resistance to repercussion and higher therapeutic dosage with increased risk of bleeding complications [5,17]. This, nonetheless, has not stopped the investigation of novel, potent and safe fibrinolytic enzymes. In this review, we will shed light on recent technological advances that facilitate the production of microbial fibrinolytic enzymes along with their properties, thrombolytic potential and additional unconventional applications.

## 2. Microbial Fibrinolytic Enzymes: Production Status and Diversity

Microorganisms exhibit a significant role in the mass-production of highly specific, low-cost fibrinolytic enzymes with feasibility of genetic modification through biotechnological approaches. During the past decades, numerous such fibrinolytic enzymes have been tested, specifically from genera *Bacillus* [2,5,14,15,18,25,26,27,28,29] and *Aspergillus* [8,19,20,30,31,32,33,34]. In addition, fibrinolytic enzymes with varied biochemical characteristics were obtained from bacterial species such as *Streptococcus hemolyticus* (Streptokinase, exudates of infected wounds) [35], *Bacillus subtilis* (Nattokinase, Fermented soybeans) [12], *Staphylococcus aureus* (Staphylokinase, human skin) [36], *Bacillus* sp. DJ-4 (Subtilisin DJ4, Doen-jang, Korea) [37], fungal species such as *Cordyceps militaris* [38] and *Armillaria mellea* [39]. Streptokinase is clinically used as an intravenous thrombolytic agent for preventing CVDs and mentioned in the World Health Organization model lists of essential medicines. It works as a plasminogen activator which forms a 1:1 stochiometric complex with plasmin, resulting in blood clot hydrolysis [40]. The fibrinolytic nattokinase initiates direct fibrin hydrolysis by converting pro-urokinase to urokinase, degrading plasminogen activator inhibitor-1 and increasing the activity of the tissue plasminogen activator. It has minimal to no after-effects when administered orally in animal/human trials and the National Science Foundation (United States) has specified its safety [12]. The fibrin-specificity of staphylokinase (SAK) is due to the rapid inhibition of the formed plasmin–SAK complex by α_2_-antiplasmin in mammalian plasma and thus allows plasminogen activation at the surface of the fibrin clot [41]. Different sources such as fermented Chungkook-jang (*Bacillus* sp. CK 11-4) [42], Dosa (*Bacillus amyloliquefaciens* MCC2606) [43], Douche (*B. amyloliquefaciens* DC-4) [44], Doen-jang (*Bacillus* sp. DJ-2, *Bacillus* sp. DJ-4) [45,46], Jeot-gals (*Bacillus* sp. KA38) [47], Gembus (*Bacillus pumilus* 2.g), [48], Kishk (*Bacillus megaterium* KSK-07) [27] and Moromi (*Bacillus subtilis* K2) [49], etc., have also been reported to yield enzymes with significant fibrinolytic potential. Probiotic lactic acid bacteria have also shown high proteolytic activity in fermented foods [50,51].

Producers of perspective fibrinolytic proteases were found among filamentous fungi (micromycetes). Some of the preparations of micromycete’ proteinases have successfully passed preclinical and clinical trials [52,53]. Effective fibrin lysis was found in proteinases from *Fusarium oxyporum* [54], *F. pallidoroseum* [55], *Paecilomyces tenuipes* [56], *Mucor subtilissimus* [57], *Rhizopus chinensis* [58] and *Rhizomucor miehei* [59]. Proteases with plasminogen activating activity were found in representatives of micromycetes of different systematic and ecological-trophic groups: *Arthrobotrys longa* [60], *Tolypocladium inflatum* [61], *Rhizopus microsporus var. tuberosus* [62], *Neurospora sitophila* [63] and *Sarocladium strictum* [64].

In addition, research suggests several other proteolytic enzymes with fibrinolytic potential from different bacterial and fungal species which are listed in Table 1.

## 3. Molecular Cloning, Overexpression and Construction of Genetically Modified Strains for Production of Fibrinolytic Enzymes

Though fibrinolytic enzymes possess significant thrombolytic potential, large production with high specificity and stability of protein is required for clinical practices, which can be achieved by techniques such as the construction of genetically modified strains, molecular cloning and overexpression, etc. In biotechnological applications, the non-pathogenicity of *B. subtilis* and its ability of exuding valuable extracellular proteins in growth medium signify its suitability as an efficient host for the expression of foreign proteins [111]. Gene encoding bacillopeptidase F was cloned from *B. subtilis* LZW, expressed in *B. subtilis* WB700 and the catalytic mechanism of proteins along with the activity of C-terminal truncation variants in sustaining enzymatic activity were interpreted [112]. A significant increase in fibrinolytic activity (80–200 urokinase U/mL) was observed when the gene expression of subtilisin DFE in *B. subtilis* was mediated by a promoter of α-amylase gene from *B. amyloliquefaciens* DC-4 [113]. Subtilisin DFE was actively expressed by vector pSUGV4 in protease-deficient *B. subtilis* WB600 in another study performed by Peng et al. [114]. Additionally, fibrinolytic gene aprE2 was overexpressed in protease-deficient heterologous *B. subtilis* [115]. One of the highest protein yields (600 mg/L of growth medium) was attained when the nattokinase gene from the *B. subtilis* strain VTCC-DVN-12-01 was expressed in eight-protease-gene-deficient *B. subtilis* WB800 under the control of promoter acoA [116]. Liu and Song also successfully achieved the cloning and functional expression of nattokinase in *B. subtilis* [117].

Some research studies report the formation of insoluble aggregates without the specific activity of enzymes when expressed in *E. coli* (Nattokinase and Subtilisin DFE) [118]. However, the successful expression of fibrinolytic enzymes in *E. coli* is also stated in literature. A 2.2-fold higher specific activity was observed when a fibrinolytic enzyme encoding Gene aprE176 was successfully cloned from *B. subtilis* HK176 and overexpressed in *E. coli* BL21(DE3) [119]. High subtilisin activity by transformant was reported when the subtilisin gene from *B. amyloliquefaciens* DC-4 and *B. subtilis* PTCC 1023 was expressed in *E. coli* BL21 (DE3) [120,121,122]. *B. pumilus* BS15 obtained fibrinolytic gene aprEBS15 was cloned in pHY300PLK (*Bacillus*-*E. coli* shuttle vector) and overexpressed in *E. coli* BL21 (DE3). Similar results with higher fibrinolytic activity were observed by *B. subtilis* transformant harboring pHYBS15 in the same study [123]. Another novel fibrinolytic enzyme (BsfA) from *B. subtilis* ZA400 was cloned and expressed in *E. coli* [124].

Information on the cloning of micromycetes-producers of fibrinolytic enzymes is scarce and scattered, which is explained by the simplicity of the cultivation of “wild” strains, because the genes of proteolytic enzymes of filamentous fungi, like all eukaryotes, contain intron regions.

However, the case of fungus *Cordyceps militaris* fibrinolytic enzyme (CmFE) has shown that there was one open reading frame of 759 bp encoding a ‘‘pre-pro-protein” of 252 amino acids. The protease was successfully cloned and recombinant CmFE was expressed as 28 kDa extracellular enzyme in yeast *Pichia pastoris* vector (pPIC9K under the AOX1 promoter) [80].

Techniques such as mutagenesis and random screening of mutants have also been employed for the improvement of microbial strains, especially bacterial. Fibrinolytic activity was doubled in research conducted by Lai et al. through in vitro random mutagenesis by ethyl methane sulfonate [125]. In another study, ultraviolet radiation mutated strain *Bacillus* sp. SFN01 displayed higher clot lysis activity (1 unit of enzyme resulted in the complete dissolution of the blood clot) than the wild strain [126]. In a similar study by Raju et al. physical/chemical mutagenesis such as ultraviolet radiation, ethyl methane sulfonate and ethidium bromide treated *Bacillus cereus* GD 55 showed optimal production of the fibrinolytic enzyme [127]. Mutations were induced in strain *Streptomyces venezuelae* by ultraviolet rays and ethyl methane sulfonate for the enhanced production of thrombinase-fibrinolytic enzymes [74]. A combination of process optimisation, codon optimisation and gene dosage has been successfully employed for high production of fibrinolytic enzyme–fibase from marine *Bacillus subtilis*, which was expressed in *Komagataella phaffii* GS115 [66]. Another approach of directed evolution (DNA family shuffling) was used to improve the fibrinolytic activity of nattokinase from *Bacillus* natto. A mutant library was generated by shuffling three homologous genes from *B. licheniformis* CICC 10092, *B. amyloliquefaciens* CICC 20164 and *B. natto* AS 1.107. One desired mutant with approximately 2.3 times higher catalytic efficiency than that of the wild strain was obtained after three rounds of DNA shuffling. The molecular modelling analysis proposed that mutations alter the surface conformation of the substrate-binding pocket and ultimately affect the enzymatic function [128]. Thus, the listed biotechnological approaches were proved efficient enough for the optimal production of fibrinolytic enzymes with enhanced specific activity.

## 4. Process Optimisation Techniques

Culture media optimisation along with the combination of biotechnological techniques play a significant role in maximising the production yield of fibrinolytic enzymes. Research suggests both traditional media optimisation one-variable-at-a-time, and statistical optimisation approaches for the production of fibrinolytic enzymes. The one-factor-at-a-time strategy was used to optimise the fermentation conditions for production of the fibrinolytic enzyme from *Citrobacter braakii*, wherein a 5.5-fold increase in enzyme production (198.6 FU/mL) was observed from the initial medium (36.15 FU/mL) [129]. However, optimisation of media components by traditional method is enormously time consuming and costly [5]. To overcome this complexity, a statistically optimised medium is employed for designing experiments, building models and analysis of the interaction of factors for desirable responses using the minimum number of experiments in most of the research. Statistical approaches such as fractional factorial design (FFD), response surface methodology (RSM), Plackett–Burman factorial design (PB Design), central composite design (CCD), central composite rotatable design (CCRD), Box–Behnken design, Taguchi orthogonal array design, etc., are employed. Vijayaraghavan et al. achieved the optimisation of process parameters (nutrient and physical parameters such as carbon sources, nitrogen sources, salt solutions, incubation temperature, pH, inoculum, etc.) for fibrinolytic enzyme production initially by one variable approach and further optimised significant variables by the statistical two-level full factorial design method [130]. A similar approach was opted by Wu et al. in 2019, in which both single-factor optimisation followed by L_9_ (3^4^) orthogonal design, an orthogonal array of four factors with three levels, were employed to optimise the process parameters [68]. A two-fold increase in the production of fibrinolytic enzymes from *Bacillus cereus* RSA1 was observed when media was optimised using the Plackett–Burman design, response surface methodology and central composite design [5]. Such optimisation methodologies thus aid in evaluating optimum nutrient composition, comprehending the interaction between different parameters and conferring reliability, henceforth saving time and energy. A detailed list of diverse media optimisation techniques used is presented in Table 2.

In the cases of obtaining fibrinolytic enzymes of fungal origin, the most preferred strategy is the transition to the solid-state fermentation cultivation of producers. Solid-state fermentation is a method of growing filamentous fungi on the surface of solid moist particles of natural origin or inert synthetic ones. At the same time, the yield of enzymes in such a cultivation system increases significantly by 1.5 times and more [142]. For example, when cultivating *Fusarium pallidoroseum* on wheat bran, it was possible to obtain an increase in the secretion of fibrinolytic protease by 1.58 times [55], and when cultivating *Aspergillus ochraceus* on silica gel and vermiculite specific activity was 1.5–3.5 times higher than in a submerged culture [143,144]. The high impact in such types of cultivation on fibrinolytic activity optimisation effect different parameters such as time, inoculum ratio, moisture content and particle size. For *Penicillium chrysogenum* SGAD12 grown on rice chaff the fibrinolytic activity was maximized at 104 h, 7% (*v/v*) inoculum ratio, 35–45% (*v/w*) moisture content and 500 µm particle size [145].

Another way to increase the secretion of fibrinolytic enzymes can be considered immobilisation. Studies carried out with microscopic fungi have shown the promise of this method, for example, culture *Penicillium chrysogenum* H9 demonstrated stable and high enzyme production in Ca-alginate beads in comparison to free cells [146]. The immobilised cells of *Aspergillus ochraceus* VKM-F4104D mycelium demonstrated increased stability and were able to synthesise fibrinolytic protease at the maximum level for 10 or more days. In addition, it was shown that immobilised mycelial cells of the producer during cultivation can be used repeatedly (up to five cycles) [147].

## 5. Recovery and Purification of Fibrinolytic Enzymes

In vitro obtained crude fibrinolytic enzymes might be employed for commercial applicability. However, the fermented culture of microbial biomass when harvested is exposed to extreme variation in environmental conditions which leads to contaminated recovery and fails to maintain the activity/stability of enzymes. Therefore, purification of enzymes is objectified to render them contaminant-free and upsurge their shelf life/stability [148]. Furthermore, biochemical properties (kinetics of clot dissolution, thermodynamic studies, etc.) and a better understanding of the structural-functional aspects of fibrinolytic enzymes can only be achieved in its pure form [5,28]. Additionally, formulations for commercial and therapeutic applications are designed only from purified enzymes [149]. Continuous disc centrifuges and vacuum drum filters are generally used for the exclusion of contaminants such as microbial cells, colloids and solids from the fermentation broth [5,150,151,152,153]. Chemical treatment of the fermentation broth and the addition of flocculating/coagulating agents is implemented to remove colloidal solids, prevent any significant loss in enzymatic activity and clogging of filters [154]. Extracting agent: Isooctane (50 mM) for fibrinolytic enzyme (1 mg/mL) in 20 mM/L at 240 rpm, 20 °C and pH 4–7 significantly aided in maximum specific activity [155]. Microbial cell-free extract is also concentrated to obtain proteins in precipitated form by techniques such as ethanol precipitation, ammonium sulphate precipitation, acetone precipitation, dialysis, ultracentrifugation and ultrafiltration [5,9,156,157,158,159,160,161]. Such methodologies are used either individually or in combinations, accompanied by chromatographic techniques for further purification. Chromatofocusing, fast protein liquid chromatography, high performance liquid chromatography, affinity column chromatography, gel filtration chromatography, ion exchange chromatography and hydrophobic interaction chromatography are commonly employed techniques for fibrinolytic enzyme purification [2,5,100,101,102,103,104,105,106,107,108,110,162,163,164,165,166,167,168]. Some recent purification studies of microbial fibrinolytic enzymes employed by researchers are discussed below. A novel fibrinolytic enzyme from *Streptomyces radiopugnans* VITSD8 was purified using solid ammonium sulphate (0–85%) precipitation accompanied by dialysis against Tris–HCl buffer (10 mM, pH 7.2) and concentrated with Millex syringe filter-ultrafiltration membrane. The enzyme was further purified using gel filtration (Sepharose CL-6B column, 120 cm × 2.2 cm) and a Poros-HQ ion exchange column (10 cm × 1 cm). A 22.36-fold increase in specific activity (3891 U/mg) of the purified enzyme was observed with a yield of 35% relative to crude enzymes [71]. A successful combination of purification techniques was reported by Hu et al. for purification of a highly potent and novel fibrinolytic enzyme DFE27 from *Bacillus subtilis* DC27 screened from Douchi. The enzyme was purified by ammonium sulphate (40–70%) precipitation, overnight dialysis (20 mM Tris-HCl buffer, pH 8.8), UNOsphere Q column chromatography, gel filtration (Sephadex G-75) and high-performance liquid chromatography. DFE27 was 69.1-fold purified (11274.4 IU/mg) with a recovery rate of 12.73% [2]. Bacifrinase from *Bacillus cereus* was well purified with ice-cold acetone (70%) precipitation and fast protein liquid chromatography (HiLoad Superdex 75 pg 16/60). An 18.3-fold purification with specific activity of 52.4 U/mg was observed [28]. Maximum 21.2-fold purification with specific activity of 2607.8 U/mL was obtained on the subsequent purification of fibrinolytic protease from *Bacillus cereus* with an anion exchange column (DEAE-sepharose) [169].

Another method for in-laboratory purification of fibrinolytic enzymes is isoelectrofocusing. The method was used for obtaining pure enzymes for their characterisation and further special studies [20,30,32,64,99]. Isoelectrofocusing allows one not only to isolate enzymes, but also to get a primary idea of their properties and compare fibrinolytic enzymes from different producers. Thus, a comparison of *Aspergillus ochraceus* L-1 and *Aspergillus terreus* 2 proteases showed that cultures are promising highly active producers of proteases of different activities toward human hemostasis proteins, activation of proenzymes such protein C, factor X and displayed plasmin, and thrombin-like activities. Using isoelectrofocusing demonstrated that both enzymes differ in the isoelectric points (nearly by one unit) and by the mechanism of action [170].

However, some studies report several disadvantages associated with these complex downstream purification technologies, such as unsustainability, being time-intensive, depletion of native protein structure, declined enzyme activity and production quality. Therefore, in such cases, techniques, namely AOT (sodium di [2-ethylhexyl] sulfosuccinate)/isooctane reverse micelles system, aqueous two-phase systems (poly-ethylene glycol 4000/8000 and sodium sulfate) and three-phase partitioning (protein precipitation by mixture of t-butanol and ammonium sulfate) are used. Liu et al. purified nattokinase by AOT (sodium di [2-ethylhexyl] sulfosuccinate)/isooctane reverse micelles system with up to 80% activity recovery and a purification factor of 3.9 [171]. Fibrinolytic enzymes from *B. subtilis* DC-2 and *M. subtilissimus* UCP 1262 were well purified by aqueous two-phase systems (polyethylene glycol/sodium sulfate) [172,173]. Henceforth, numerous purification methodologies opted for in research studies on fibrinolytic enzymes have significantly enhanced their specific activity with a high recovery rate and can be further formulated for commercial/therapeutic practices.

## 6. Physicochemical Characterisation of Microbial Fibrinolytic Enzymes

Physiochemical properties such as optimal pH/temperature, molecular mass, effect of metal ions/inhibitors and substrate specificity of fibrinolytic enzymes have been extensively studied. Table 3 summarises important characteristics of microbial fibrinolytic enzymes. Most of the reported fibrinolytic enzymes are active at neutral to alkaline pH with optimal activity amid pH 7 to 10 [2,5,8,19,62,63,64,104,174,175,176], while some fibrinolytic enzymes possess optimal specific activity at extreme acidic or basic conditions. Fibrinolytic enzymes from *Flammulina velutipes* and *Pseudomonas baetica* SUHU25 were found to exhibit optimal activity at pH 6 [158,177]. Nattokinase extracted from mutant *Pseudomonas aeruginosa* CMSS obtained its highest activity at pH 5 [159]. Fibrinolytic enzymes from fermented shrimp paste and *Staphylococcus* sp. strain AJ from Korean salt-fermented Anchovy-jeot exhibited optimal activity from pH 3.0–7.0 and pH 2.5–3.0, respectively [178,179]. Optimum pH for the activity of fibrinolytic enzymes CFR15 and CK from *B. amyloliquefaciens* MCC2606 and *Bacillus* sp. strain CK 11–4, respectively, was found to be pH 10.5 [42,43]. Fungal enzymes, such as *Aspergillus ustus* 1, *Arthrobotrys longa* 1 and some others are able to hydrolyse fibrin at pH 6.0 [30,176]. Optimal temperature widely ranged from 20–70 °C [20,31,42,100,101,177,180], mostly approx. 50 °C for bacterial proteases [5,181] and 37–45 °C for some fungal species [20,30,62,101,104,107]. The molecular weight of fibrinolytic enzymes of different origins broadly varied from 14 kDa to 97 kDa [56,140,172], mostly within 27 kDa and 44 kDa [5,9,182,183].

The effect of metal ions/inhibitors on the fibrinolytic activity of enzymes depends on its nature of action, i.e., serine protease, metalloprotease or serine metalloprotease. A wide range of metal ions (Na^+^, Ag^+^, K^+^, Fe^2+^, Ti^2+^, Mg^2+^, Zn^2+^, Ni^2+^, Co^2+^, Ca^2+^, Mn^2+^, Cu^2+^, Hg^2+^, Al^3+^, Fe^3+^) have been studied for their effect on the biological activity of enzymes. The activity of few fibrinolytic proteases was influenced by divalent metal ions like Mg^2+^, Fe^2+^, Mn^2+^, Mg^2+^, Ca^2+^ and inhibited by Cu^2+^, Fe^3+^, Zn^2+^, Hg^2+^, Al^3+^, [56,62,63,163,184]. Some fibrinolytic metalloproteases require divalent ions Co^2+^, Ni^2+^, Zn^2+^ for their activity [42,185]. Among inhibitors, Di-isopropyl fluorophosphate (DFP) and phenyl methyl sulfonyl fluoride (PMSF) are the frequently used irreversible serine protease inhibitors [5,19,20,27,30,31,32,63,92]. Inhibitors such as ethylene diamine tetra acetic acid (EDTA), ethyleneglycol bis(2-aminoethyl ether)-*N,N,N’,N’*-tetraacetic acid (EGTA) and 1, 10 phenanthroline strongly inhibit the fibrinolytic activity of metalloproteases [73,163,183]. In contrast, the fibrinolytic activity of the third ‘serine metalloprotease’ class of enzymes is inhibited by both serine and metalloprotease inhibitors [22,23,58,104].

The action of purified fibrinolytic enzymes has been studied on various natural protein substrates such as fibrin, fibrinogen, gelatin, casein, bovine serum albumin (BSA), keratin, collagen, globulin, azoalbumin and haemoglobin [23,30,32,90,99,130]. The high substrate specificity of fibrinolytic enzymes is testified towards fibrin, which is a more distinctive feature than other reported proteases with broad substrate specificity. Fibrinolytic enzyme CK produced from *Bacillus* sp. strain CK11-4, screened from Chungkook jang possesses an 8-fold higher specific activity towards fibrin than substilisin Carlsberg, an alkaline protease with a similar *N*-terminal sequence [42]. In another study, the ratio of specific fibrinolytic to caseinolytic activity of subtilisin DJ-4 from *Bacillus* sp. DJ-4 was 3.97 and 2.67-fold higher than subtilisin Carlsberg and subtilisin BPN, correspondingly [37]. For some filamentous fungi, it was shown that the ratio of overall proteolytic (non-specific) activity to fibrinolytic activity is one of the effective fibrin hydrolysis criteria and it is within the values 0.18–0.57 [186].

Microbial fibrinolytic enzyme specificity has been spectrophotometrically assessed by deploying synthetic substrates D-Val-Leu-Lys-pNA, N-Benzoyl-Pro-Phe-Arg-p-NA, H-D-Val-Leu-Lys-pNA (plasmin substrate), MeO-Suc-Arg-Pro-Tyr-pNA, N-succinyl-Ala-Ala-Pro-Phe-p-NA, (chymotrypsin/subtilisin substrate) and D-Val-leu-Arg-pNA (kallilrein substrate), pyro-Glu-Gly-Arg-pNA (urokinase substrate), H-D-Phe-Pip-Arg-pNA, N-Benzoyl-Phe-Val-Arg-pNA, Tos-Gly-Pro-Arg-pNA (thrombin/trypsin substrate) [2,27,42,48,177,180]. Fibrinolytic enzymes QK-1 (plasmin-like serine protease) and QK-2 (subtilisin-family serine protease) obtained from *Bacillus subtilis* QK02 exhibited higher activity towards H-D-Val-Leu-Lys- pNA and N-succinyl-Ala-Ala-Pro-Phe-p-NA, respectively [78]. Fibrinolytic enzymes DFE27 (tissue-type plasminogen activator) from *B. subtilis* DC27 and AO-3 from *Aspergillus ochraceus* VKM F-4104D showed specificity towards plasmin substrate D-Val-Leu-Lys-pNA [2,20]. A subtilisin-like fibrinolytic enzyme from *Lactobacillus plantarum* KSK-II hydrolysed Suc-Ala-Ala-Pro-Phe-pNA [187]. A fibrinolytic protease secreted by micromycete *Sarocladium strictum* was able to actively split off a chromogenic peptide substrate pGlu-Gly-Arg-pNA [26].

Details of various physiochemical properties of bacterial and fungal fibrinolytic enzymes are mentioned in Table 3.

## 7. Structural-Functional Attributes

An evaluation of structural-functional characteristics and mechanisms of action is imperative for the massive applicability (industrial/therapeutic) of enzymes [24,203]. However, literature reports limited research on structural-functional attributes and intermolecular interactions of fibrinolytic enzymes [24]. In a recent study, homology structural modelling servers Iterative threading ASSEmbly Refinement (I-TASSER), RaptorX, Protein Homology/analogY Recognition Engine V 2.0 (Phyre2) and SWISS-MODEL were used to model a three-dimensional (3D) structure of fibrinolytic protease RFEA1 from *Bacillus cereus* RSA1, and structural validation was accomplished by structural analysis and verification server (SAVES v6.0). In addition, structural modelling revealed the presence of a high-affinity calcium binding site (Ca1), associated with hydrogen bonds at Asp^147^, Leu^181^, Ile^185^ and Val^187^ RFEA1 residues. Furthermore, an enzyme-substrate docked complex (RFEA1-fibrin) exhibited a high binding affinity (−21.36 kcal/mol), suggesting the significant activity/specificity of enzyme and serine (subtilisin) catalytic residues were observed (Asp^146^, Ser^164^ and His^132^) [24]. Subtilisin enzymes have high-affinity (Ca1) and low-affinity (Ca2) calcium binding sites which play vital roles in the thermostability of enzymes and prevent autolysis. Enhanced thermostability due to the presence of calcium binding sites was reported for *Bacillus subtilis* HK176 produced fibrinolytic enzymes (AprE176: 11% and M179: 36%) [119], while a significant activity increase (122.02 ± 5.71%) was observed for *Bacillus subtilis* DC27 produced fibrinolytic enzyme in the presence of 5 mM Ca^2+^ ions [2]. Another study reported on the I-TASSER modelled structure of a serine fibrinolytic protease Bacifrinase which entailed the catalytic triad of Asp^102^, His^83^ and Ser^195^. The bacifrinase–fibrinogen (Bβ-chain) interaction was stable with a geometric shape complementarity score of 19698, interface area of 2522.80 and ACE of 442.39 [28]. Further, the SWISS-MODEL predicted a 3D structure of subtilisin K2 when it underwent docking using the High Ambiguity Driven protein–protein DOCKing (HADDOCK) webserver against substrate fibrin and showed a Kd value of 6.3 · 10^−15^ M and binding affinity of − 19.4 kcal/ mol. Structural superimposition of subtilisin K2 on nattokinase generated a root mean square deviation of 0.12 Å and indicated a significant similarity between the two proteins. However, the positions of active site residues of subtilisin K2 (Asp^19^, His^51^, and Ser^208^) were dissimilar from nattokinase active site residues (Asp^32^, His^64^, and Ser^221^) [49]. A detailed list of the structural-functional attributes of microbial fibrinolytic enzymes is listed in Table 4. 

## 8. Thrombolytic Potential of Microbial Fibrinolytic Enzymes

In vitro and in vivo estimation of the thrombolytic potential of fibrinolytic enzymes significantly aid in their potential use in clinical practices and hence commercialisation. There are varieties of methods proposed to access the in vitro anti-thrombotic potential of enzymes using blood and its elements, which provide indispensable inputs before animal modelled studies [209].

### 8.1. In Vitro Fibrinolytic Assays

There are numerous methodologies adopted for the assessment of the clot lysing potential of enzymes, for example, fibrin plate assay, fibrin micro-plate assay, rapid fibrin plate assay, euglobulin clot lysis time, global fibrinolytic capacity and viscoelastic methods, etc [210,211,212,213,214,215]. Fibrin/clot lysis outcome in such methods is generally measured by evaluating the zone of hydrolysis, calorimetric methods and nephelometry [5,216,217]. Our review enlightens different assays used by researchers in detail. The fibrin plate assay is a delicate and detailed process for determining fibrinolytic mediators. The assay comprises of either fibrin clot as a substrate (formed by the addition of thrombin to fibrinogen) or fibrin directly. It is usually performed in two systems. The first being the ‘plasminogen free fibrin plate assay’, is entitled for the direct action of plasmin-like enzymes. Endogenous fibrinolytic factors such as plasminogen and plasmin are deactivated at high temperature conditions (mostly 80 °C for 30–45 min) [5]. The second is ‘plasminogen rich fibrin plate assay’, which is not exposed to extreme temperature conditions and is suitable for plasminogen activators [16]. A certain amount of difficulty and uncertainty in determining the lysis zone was observed in fibrin plate assay and to overcome this problem fibrin micro-plate assay was developed. It is a high capacity sensitive and quantitative fibrinolytic micro-technique in which fibrin clots are moulded in wells of immense adsorption microtiter plates with suitable dye integrated into them with the help of fibrinogen. For reference, serial dilutions of urokinase/plate (standard) are added into these microtiter plates. Inhibitors are removed from citrated test plasmas before their application into the wells through acetone treatment. After suitable incubation, the lysate formed is discarded and fibrin is photometrically determined after dissolution by plasmin. The advantage associated with this assay is the reliable degree of clot lysis assessment by adjusting incubation time and varying concentration of standard urokinase [218].

A long incubation period was the major drawback of fibrin plate assay which was improved by rapid fibrin plate assay carried out to inspect plasminogen enrichment. In a study conducted by Marsh and Gaffney, fibrin plates were modified by the addition of two casein units of plasminogen to form firm and opaque plates for clarity. The clots formed did not lyse impulsively and yielded effective parallel lines for urokinase and streptokinase after an incubation period of 3 h instead of 16–20 h [212]. Another in vitro euglobulin clot lysis time (ECLT) assay is used to evaluate plasma fibrinolytic capacity and signifies the interaction of activity amongst the tissue plasminogen activator and plasminogen activating inhibitor [219]. It measures the change in optical density of the recalcified euglobulin fraction present in plasma samples over different time intervals [213]. A quantitative ECLT assessment is performed by a microtiter plate reader providing reliable and reproductive data where lysis time is determined by the midpoint between minimum and maximum turbidity. The turbidity is measured by the microtiter plate reader after every 30 min. Its mathematical examination not only determines critical points of lysis curvature but also analyses kinetics of fibrinolysis. It is used for determining hyperlipemic condition, atherosclerosis and its associated diseases, cardiovascular surgery, pharmacological surgery and liver transplantation coagulation surgery. Global fibrinolytic capacity (GFC) is used to analyse single sample, evaluating fibrinolysis by generation of D-dimers (DD) from the fibrin clot [219]. In this assay, the fibrin clot was prepared with plasminogen free fibrinogen and thrombin. It was allowed to freeze dry and used to make fibrin tablets (25 µg) with silica. These tablets were added to tubes containing platelets poor plasma and tPA. After incubation at 37 °C for one-hour, the fibrinolytic process was reduced by the accumulation of aprotinin and the generation of D- dimers were measured [209]. It is an expensive assay due to the DD evaluation and reagents used [219]. GFC is used to estimate fibrinolysis activity (plasma) in various conditions such as diabetes type I and II, sepsis, polycystic ovary syndrome, chronic liver disease, mitral valve disease, respiratory distress syndrome and hypothyroidism [209].

However, the above-mentioned traditional approaches of thrombolysis assessment encompass direct experimental manual handling, which might introduce errors and thus limit precision. In such cases, rheology methods (thromboelastometry, thromboelastography and sonoclot) based on the viscoelastic properties of blood clot formation and dissolution are employed. Viscoelastic methods are used to analyse clotting and fibrinolytic processes in whole blood and examine the influence of blood cells and platelets. The major advantages associated are: speed and the important role in point of care tests (POC) during surgical procedure related to blood loss, liver transplant, traumatic injury and cardiothoracic surgery [220]. The first method ‘thromboelastometry (ROTEM)’ computes the viscoelastic properties of clot, kinetics of clot growth, provides data on speed of coagulation commencement, clot firmness and breakdown [221]. It involves differential testing on one patient using four channels. This testing involves Intem (Intrinsic coagulation with ellagic acid), Extem (Factor triggering extrinsic activators), Fibtem (Cytochalasin D added to Extem to eliminate platelet role) and lastly Aptem (Aprotinin added to Exten to stop fibrinolysis). Temograms are used to display all results of clotting and lysis curves [220]. Different parameters such as clotting time in seconds, amplitude 10 (clot amplitude 10 min after the commencement of the clotting in mm) and maximal clot firmness (mm) can be recorded from ROTEM tests. The second ‘thromboelastography (TEG)’ involves various tests possessing differential reagents such as kaolin instead of ellagic acid in Intem [220]. It can quickly determine low coagulation, hypercoagulation or solidification to fibrinolysis (involving prothrombin, thrombin, fibrin formation, stability and elasticity of blood clot) in the patient’s blood directly. TEG involves the Estimated Percentage of Lysis (EPL) or Lysis at 30 min (LY30) [222]. TEG and ROTEM analysers evaluate the actual properties of a clot by the use of an immobile cylindrical cup holding the citrated whole blood and calcium chloride with specific activators and oscillating at an angle of 4°–45°. Each rotation cycle persists for 10 s. A pin is suspended vertically in the blood through a torsional wire and is supervised according to the motion. The torque of the revolving cup is conveyed to the submerged pin only after the fibrin platelet bonding links the cup and pin together. The magnitude of the pin motion is affected by the bonding of these fibrin platelets. Hence, the strength of the fibrin platelet is directly linked with the magnitude of the output. When these clots are broken down during lysis the cup motion is reduced. The rotational motion of the pin is transformed by an automatic electrical transducer to an electrical signal leading to a graphical display (plot showing viscoelastic properties of the clot over time) which is supervised by a computer [221,222]. The third alternative ‘sonoclot’ measures the change in resistivity to the movement on a small probe pulsating at an ultrasonic frequency in a coagulating blood sample. The resistivity is induced by the emerging clot. An open ended, hollow disposable plastic probe mounted on an ultrasonic transducer which trembles vertically at a rate of 100 Hz (distance of 1µm) is submerged in a cuvette comprising blood sample (0.4 mL) at fixed depth. The cuvette applies a viscous drag on the probe and this dragging force upsurges as the sample clots. Fibrin components form on the tip of the probe and between the probe and wall of the cuvette, eventually enhancing the mass of the probe. This increase in resistivity to the vibration of the probe due to the clotting of the sample is sensed via electronic circuits which are then transformed to an output signal on a paper chart plotter reflecting the viscoelastic properties of the clot. This unceasing output signal describes the blood coagulation process starting from fibrin development, aggregation of fibrin monomers, platelet interaction, clot retraction and lastly lysis [223].

Nevertheless, these viscoelastic techniques also have limitations such as insufficient equipment accessibility, number of tests executed at a time (extreme four samples) and required sample (whole blood) size (300 μL). Therefore, researchers have developed and suggested a high throughput whole blood thrombolysis plate assay to overcome the limitations of existing techniques. The halo assay technique by Bonnard et al. includes the formation of halo shaped, tissue factor prompted, whole blood clots in 96-well plates. The clot lysis rate using different doses of plasmin, urokinase and tissue plasminogen activator (t-PA) was determined with a spectrophotometer plate reader. Results revealed that plasmin directly acted on halo-clots with a short activation time whereas t-PA displayed a distinct lysis protocol, which might be due to the conversion of endogenous plasminogen into plasmin and then fibrin degradation. The activation time was significantly delayed with urokinase, and the utmost rate of clot degradation was reduced on aged clots. The technique overcomes limitations by a considerable reduction in the required volume of blood and imparting high throughput screening on a whole blood-based assay. Furthermore, the methodology discovered could significantly be transformed to clinical practices as a point-of-care assay to upgrade the diagnosis of cardiovascular disorders [224]. Such techniques have also been used for the evaluation of the thrombolytic potential of thrombin-activatable microplasminogen and thrombin-degradable capsules [225,226].

### 8.2. In Vivo Thrombolytic Assays

An in vivo/animal modelled study is an essential step to comprehend the pathophysiology of cardiovascular thrombosis and function as an efficient platform to assess novel therapeutics for the prevention and cure of thrombolytic complications. The fibrinolytic enzymes under in vivo examination are either administered intravenously or as oral anti-coagulants. Our review discusses details of all reported in vivo models, both based on thrombus initiation/lysis and indirect measurement of fibrinolysis (D-dimer test, carrageenan-induced thrombosis model, ferric chloride-induced thrombosis model, laser-induced and phytochemical injury).

The first ‘D-dimer assay’ monitors fibrin lysis by detecting the presence of D-dimer fragments released in mammalian blood by immunoassays (immunoturbidimetry, latex agglutination and enzyme-linked immunosorbent assay) with monoclonal antibodies specific for D-dimer domain. It determines the duration of anti-coagulation therapy and is expressed in mass units: D-dimer unit (DDU) at 195 kDa and fibrinogen equivalent unit (FEU) at 340 kDa [227]. The test is employed during pulmonary embolism, arterial and venous thromboembolism [228,229,230] but lacks in reproducibility, non-applicability for fibrinogenolysis (as D-domain is not present in derivatives of fibrinogen) and variation in type/magnitude of D-dimer units which leads to confusion [227]. Further, the comparatively reliable carrageenan-induced thrombosis model has been extensively employed for testing of numerous anti-thrombotic agents clinically [14,75,231,232,233,234]. Carrageenan is a mucopolysaccharide obtained from edible red seaweeds, encompassed by repeating units of sulphated/nonsulphated anhydrogalactose and galactose connected by glycosidic linkages. On the basis of the different positions/numbering of sulphur groups on galactose units, three carrageenan classes, kappa, iota and lambda carrageenan are available [235]. However, k-carrageenans (kappa) were stated to be of the utmost significance [236] and were used in the majority of the experiments [13,75,234,237]. The thrombolytic agent to be tested is injected intraperitoneally followed by intravenous injection of sterilized carrageenan and thereafter, animal tail is submerged in ice water to observe the appearance/dissolution of a wine-coloured clot [233]. Major advantages associated with the use of this model include: precise evaluation of thrombus dissolution and drug efficacy, need of fewer animals without killing them and evading of complex surgery exposing blood vessels in animal tails. Additionally, k-carrageenan may impact the deactivation of Hageman factor [238], which is followed by endogenous coagulation [239].

The ferric chloride-induced thrombosis model has also been extensively used to assess the in vivo efficacy of anti-platelet drugs and anti-coagulants [14,239,240]. Intravascular thrombus is introduced by the application of ferric chloride into an intact vessel (for example: carotid artery) [14] and the effectiveness of the test compound is examined. The thrombolytic agent which is to be tested is introduced intraperitoneally into the vessel followed by a ferric chloride solution. Doppler flowmeter reflecting the occlusion time is used to monitor the blood flow in the vessel. In addition, observations can be studied using intravital microscopy as well. The time period from the initial injury to complete vessel destruction is the measured parameter [241]. The clot formation induced into the vessel is closest to the human pathological condition and is a major merit associated with this assay. Furthermore, the model is sensitive to various thrombin inhibitors, representing its reliability for in vivo assays. However, diagnosis and treatment of deep vein thrombosis (DVT) must be performed in combination with other assays as the entire vessel injury predicts a lesser number of DVT [242]. In fact, alternative strategies for in vivo thrombus formation such as laser-induced and phytochemical injury have also been employed in most studies to determine the thrombolytic potential of the test compound. The laser-induced injury model is based on the induction of thrombus through heat damage to a defined endothelium section [243], whereas, in a phytochemical injury a photosensitizing dye (for example: rose bengal) is used, which causes photo-excitation and leads to oxidative damage to the wall of the blood vessel, ultimately resulting in thrombus formation. The thrombolytic agent to be tested is administered prior to thrombus formation in both the mentioned models. Advantages associated are: (i) the damage caused to endothelium by laser-induced injury is more limited than the ferric-chloride induced method which results in the complete denudation of endothelium [243], (ii) lower systemic toxicity and increased phytochemical efficacy are added merits of the phytochemical injury technique [244].

Moreover, the thrombolytic effect of nattokinase has been investigated on clots introduced by acetic acid injury in the carotid artery of a rat. A 62% recovery rate of arterial blood flow was observed in rats treated with nattokinase in comparison to plasmin with 15.8% recovery and elastase with no recovery [245]. Oral administration of nattokinase has been investigated and clinically tested in both animal and human trials. Research testifies the efficacy/stability of nattokinase in mammalian digestive tracts with continued action (8–12 h) and the prevention of thrombus formation with an oral daily dose of nattokinase (2000 FU or 50 g) [12]. In another study by Sumi et al. in 1990, the oral administration of four capsules of nattokinase completely dissolved blood clots (within 5 h) administered in the leg vein of dogs [246]. Human trial with 12 healthy participants when administered with 200 g/day of nattokinase enhanced the blood clot dissolution ability of volunteers even after 2–8 h of administration [22]. Urano et al. reported the ability of nattokinase in the cleavage of PAI-1 (primary fibrinolysis inhibitor) [247]. Henceforth, several microbial fibrinolytic agents have been subjected to in vivo animal/human trials to this day.

## 9. Other Miscellaneous Applications of Microbial Fibrinolytic Enzymes

Along with clot lysis efficacy, microbial fibrinolytic enzymes have been reported to exhibit many other miscellaneous applications in clinical, industrial and food sectors such as blood pressure regulation, proteolysis in addition to fibrin, detergent additives, antimicrobials and anti-inflammatory agents, etc. A detailed review of reported prodigious applications of microbial fibrinolytic enzymes is discussed below.

### 9.1. Microbial Fibrinolytic Enzymes in Clinical Practices and Food Sector

A recent in vivo study conducted on rats reported the use of nattokinase-like enzyme (NK-01) in thrombosis along with the reduction of blood pressure and prevention of atherosclerosis. Label-free liquid chromatography–tandem mass spectrometry technique was employed to examine NK-01 effects on the proteomic profiling of plasma proteins. NK-01 inhibited angiotensinogen translation to AngII, thus promoting kininogen lysis to control blood pressure. The protease was also said to increase the amount of paraoxonase 1 to thwart atherosclerosis [248]. Kim et al. also performed a randomised human trial to study the effects of nattokinase on blood pressure/hypertension. A total of 86 volunteers (20 to 80 years) with systolic blood pressure (130 to 159 mmHg) were fed with nattokinase (2000 FU/capsule) for 8 weeks. The outcome confirmed that the intake of nattokinase resulted in reducing systolic and diastolic blood pressure [249]. Oral administration of nattokinase and serrapeptase is reported to show a protective effect against Alzheimer’s disease [250]. Enzyme serrapeptase is stated to have potent anti-inflammatory and other beneficial properties along with thrombolytic activity [6,16,251,252]. Panagariya and Sharma examined the response of serrapeptase in patients with carpal tunnel syndrome (CTS). Twenty patients were administered with serratiopeptidase (10 mg twice/daily) and results were evaluated after 6 weeks. Significant improvements in sixty-five percent of cases without any after-effects were observed [253]. The efficacy of serrapeptase has also been tested and confirmed in the treatment of venous inflammatory disease, antiedemic activity, inflammation in patients with breast engorgement, chronic airway diseases (decreased neutrophil count, sputum output and viscosity) and chronic sinusitis, etc. [254,255,256,257]. In another in vivo animal trial, serrapeptidase was found effective against *Staphylococcus epidermidis* in the abolition of infection with increased antibiotic efficacy [258]. Furthermore, in a study conducted in men with amicrobial prostato-vesiculitis, serrapeptidase along with other anti-inflammatory drugs efficiently reduced the swelling of prostate glands [259]. An amino acid sequence of Ace02 (fibrinolytic enzyme from *B. vallismortis* Ace02) exhibited strong sequence similarity with L27 (bacteriolytic enzyme from *B. licheniformis*), which has potent lytic potential against the pathogen of dental caries (*Streptococcus mutans*), thus suggesting its use for the prevention of dental caries along with thrombosis [260].

Wu and Xu reported that fibrinolytic protease of *Fusarium* sp. CPCC 480097 (Fu-P) in a rat model of artery-vein bypass thrombosis might also be used as a natural agent for thrombolytic therapy or thrombosis prevention [53]. The intravenous injection of Fu-P produced a 58.4% inhibition ratio of thrombus formation at 0.1 mg/kg body weight, compared with heparin which produced a 42.5% inhibition ratio of thrombus formation at 0.6 mg/kg body weight. Fu-P also significantly prolonged fibrinogen clotting time, activated partial thromboplastin time and thrombin time. It was shown that the protease was not the inhibitor of the thrombin and Xa [53]. Another fungal fibrinolytic protease of *Mucor subtilissimus* UCP 1262 was tested in the experiments on *Mus musculus* and can be regarded as a potential competitor for developing novel anti-thrombotic drugs. The assay to assess blood biocompatibility shows that at a dose of 0.3–5 mg/mL the haemolytic grade is considered insignificant. It was shown that this enzyme did not prolong bleeding time in mice when dosed with 1 mg/kg [52].

Fibrinolytic enzymes have also been reported with a momentous role in food fortification and nutraceutical applications, suggesting their use in reducing the risk of cardiovascular diseases [261]. A novel fibrinolytic enzyme from fermented shrimp paste (Asian fermented seasoning) was reported with significant potential for food fortification and nutraceutical applications along with its use in thrombolytic therapy [178]. Fibrinolytic alkaline protease KSK-II from *Lactobacillus plantarum* KSK-II was found to inhibit the growth of *S. aureus* (29%), *B. cereus* (21%), *P. aeruginosa* (13%), *P. vulgaris* (10%), *E. coli* (7%) and *K. pneumonia* (0%). The anti-fungal activity of KSK-II was observed against *Rhizoctonia solani* (soilborne plant pathogen) [187]. Such proteases are applied as anti-microbials for wall perforation to release endogenous metabolites [262] and in food and medical applications [263].

### 9.2. Industrial Applications of Microbial Fibrinolytic Enzymes

Some fibrinolytic enzymes exhibit a wide spectrum for proteolysis in addition to fibrin. KSK-II (fibrinolytic enzyme from *L. plantarum*) was found to hydrolyse plasma proteins along with collagen and fibrin. This attribute of KSK-II prohibited its use in in vivo thrombus hydrolysis as the action of enzyme on haemoglobin and collagen might lead to haemorrhage. KSK-II was also compatible and stable with detergent formulations Persil (112%), X-tra^®^ (98%), Ariel^®^ (92%), Tide^®^ (86%), Lang^®^ (81%), Dac^®^ (80%), Isis^®^ (77%), Bonux^®^ (75%), Dixan^®^ (67%) and Oxi^®^ (64%). Optimal pH and temperature of the enzyme was pH 10.0 and 50 °C with wide stability at pH 7.5–12.0 and up to 70 °C. Thus, the enzyme was considered a suitable detergent additive [187]. Masilamani and Natarajan reported a fibrinolytic enzyme from *Marinobacter aquaeolei* MS2-1 with significant activities with detergents Ujala, Tide, Wheel, Surf, Excel and Kite and maximum enzyme activity with Rin [264]. An increase in activity (up to 141%) of a detergent-resistant nattokinase from *B. subtilis* VTCC-DVN-12-01 was observed with non-ionic detergents (Tween-20, Tween-80 and Triton X-100) [116]. A fibrinolytic enzyme from *Bacillus* sp. IND12 hydrolysed egg white, chicken skin, goat blood clot and bovine serum albumin, which recommended its use in both clinical practices and wastewater treatment [130]. Henceforth, microbial fibrinolytic enzymes with blood clot dissolution efficiency might be explored for potential applications in industrial sectors as well.

## 10. Conclusions

In conclusion, our review discusses in detail the diverse sources, optimisation techniques, production (isolates and mutants/recombinants), in vitro/in vivo thrombolytic trials and tremendous possibilities of microbial fibrinolytic enzymes towards therapeutic (blood clot removal/treatment of cardiovascular thrombosis) and food/detergent industry deployment. The high efficacy/specificity of fibrinolytic enzymes from fermented foods like Chungkook-jang, Dosa, Douche, Doen-jang, Jeot-gals, Gembus, Kishk, Moromi, etc., was observed, when equated with other sources (culture collections, milk, soil samples or marine isolates). Furthermore, techniques such as the construction of genetically modified strains, molecular cloning/overexpression and mutated sources significantly enhanced the fibrinolytic potential, specificity/stability and scale up of the enzymes. Optimal production of enzymes was achieved through statistically optimised media, thus eradicating the enormous substrate cost and time. Most of the fibrinolytic enzymes have been successfully purified and characterised to evaluate their physiochemical properties, functional moiety and mechanism of action. Furthermore, in vitro and in vivo studies of thrombolytic assays alone or in combinations have detailed the clinical effectiveness and safety of enzymes, alleviating the comprehensive pathophysiology of thrombosis in mammals. In addition, surplus applications such as blood pressure regulation, anti-microbial/anti-inflammatory potential, detergent additives, etc., of some fibrinolytic enzymes are reported. However, the mechanism behind the aforementioned applications is so far uncertain.

## Figures and Tables

**Table 1:** Microbial sources of fibrinolytic enzymes.

Collection Site/Source	Microbial Strain	Fibrinolytic Enzyme	Reference
Indonesian fermented soybean	*B. cereus* K1, *B. subtilis* K2, and *B. cereus* K3	-	[65]
Marine isolate	*B. subtilis* D21	-	[66]
Fermentation with combinations of microbial strains	*B. subtilis* GUTU09, *Lactobacillus* sp., *Bifidobacterium* sp. and *Mucor* sp.	Nattokinase	[67]
UV mutagenesis of *B. subtilis* HQS-3	*B. subtilis* strain WR350	-	[68]
Fermented soybean and soil	*B. subtilis*	Nattokinase	[69]
Garbage dump soil	*Serratia* sp. KG-2-1	-	[21]
Soil samples	*B. cereus* RSA1	-	[5]
Fermented food of North- East India	*B. cereus*	Bacifrinase	[28]
Marine soil	*Streptomyces rubiginosus* VITPSS1	-	[70]
Marine sponges (*Agelas conifera*)	*Streptomyces radiopugnans* VITSD8	-	[71]
Fermented food Jotgal (pickled fish)	-	-	[72]
Gut of a Colombian silkworm hybrid	*Serratia marcescens* C8	Serratiopeptidase	[73]
Marine water	*Streptomyces venezuelae*	Thrombinase	[74]
Indonesian soybean-based fermented food	*Stenotrophomonas* sp.	-	[75]
Bovine milk	*Streptococcus agalactiae* EBL-31	Streptokinase	[76]
Vietnamese soybean-fermented food samples	*B. amyloliquefaciens*	-	[77]
Fermented soyabean	*B. subtilis* QK02	QK01 and QK02	[78]
Doen-jang	*Bacillus* sp. DJ-2	Bacillopeptidase DJ-2	[45]
-	*Bacillus cereus* B80	-	[79]
China General Microbiological Culture Collection Center (2577)	*Cordyceps militaris*	CmFE	[80]
*Catharanthus roseus* (Stem)	*Xylaria curta*	Xylarinase	[81]
Korean Mushroom Company (Suwon)	*Hericium erinaceum*	Herinase	[82]
Edible mushroom	*Pleurotus ferulae*	-	[83]
Las Yungas rainforest (Tucumán, Argentina)	*Bionectria* sp.	-	[84]
Korean agricultural culture collection	*Schizophyllum commune*	Mushrokinase	[85]
-	*Aspergillus ochraceus* 513	-	[86]
Commercial rice-koji	*Aspergillus oryzae* KSK-3	-	[87]
Starter for brewing rice wine	*Rhizopus chinensis* 12#	-	[88]
Caatinga soil (Brazil)	*Mucor subtilissimus*	-	[89]
Herbal medicine	*Cordyceps sinensis*	CSP	[90]
Korean agricultural culture collection	*Fomitella fraxinea*	FFP1 and FFP2	[91]
Marine isolate	*Codium divaricatum*	CDP	[92]
Marine isolate	*Codium latum*	CLP	[93]
Marine isolate	*Codium intricatum*	CIP-I and CIP-II	[94]
Shiokara (Japanese traditional fermented food)	-	Katsuwokinase	[95]
Mutant of *Streptomyces spheroides* 35	*Streptomyces spheroides* M8-2	-	[96]
Centre of Cultures of the National Research Centre (Cairo, Egypt)	*Cochliobolus lunatus*	-	[97]
-	*Actinomyces thermovulgaris*	-	[98]
Soil isolate, nematophagus	*Arthrobotrys longa* Mecht.1	Longolytin	[60]
Microorganisms Collection of Department of Microbiology, Moscow State University	*Aspergillus flavus* O-1	-	[32]
Microorganisms Collection of Department of Microbiology, Moscow State University	*A. fumigatus* D-1	-	[99]
Regional Hospital and University of Angers	*A. fumigatus* CBS 113.26	-	[100]
Soil isolate	*A. brasiliensis* AUMC 9735	-	[8]
Soil isolate	*A. brasiliensis* BCW2		[101]
Soil near slaughterhouse	*A. carbonarius* S-CSR-0007	-	[31]
All-Russian collection of microorganisms, Moscow	*A. ochraceus* VKM F-4104D	AO-3	[20]
Soil isolate	*A. tamarii* SAS 02	-	[33]
Soil isolate	*A. terricola*	Terrilytin	[19]
Microorganisms Collection of Department of Microbiology, Moscow State University	*A. ustus* 1	-	[30]
Tectona grandis (Teak wood) infected leaf sample	*Cladosporium* sp.	-	[102]
Sodx Co., Osaka, Japan	*Fusarium* sp. BLB	FP	[103]
Chrysanthemum stems	*Fusarium* sp. CPCC 480097	Fu-P	[104]
Soil isolate	*Fusarium* sp. CSN-6	-	[105]
Soil isolate	*Mucor subtillissimus* UCP 1262	-	[106]
Starter used for fermenting soybean paste	*Neurospora sitophila*	-	[63]
Compost preparations of factory of organic fertilizers, Egypt	*Oidiodendrum flavum*	-	[107]
*Hibiscus* leaves	*Penicillium citrinum*	-	[108]
Contaminated soil of poultry slaughterhouse	*Penicillium* sp. BF20		[109]
Daqu (a fermentative agent used in the production of Chinese liquor and vinegar)	*Rhizopus microsporus* var. *tuberosus*	-	[62]
Mycophylic strain	*Sarocladium strictum*	Proteinase III	[64]
Human sputum	*Scedosporium apiospermum*	-	[110]
Insects’ remains	*Tolypocladium inflatum* k1	-	[61]

**Table 2:** Process optimisation techniques for optimal production of fibrinolytic enzymes.

Microbial Strain	Optimisation Technique	Fibrinolytic Activity (U/mL)	Reference
*Fictibacillus* sp. SKA27	CCD and artificial neural network (ANN)	4175.41	[131]
*Bacillus subtilis*	CCD	18.9	[132]
*Bacillus cereus* RSA1	PB Design, RSM and CCD	30.75	[5]
*Serratia rubidaea* KUAS001	One factor at a time approach	394.9	[133]
*Bacillus subtilis*	PB Design and Box–Behnken design	77,400	[134]
*Streptomyces radiopugnans*_VITSD8	One factor at a time and Fractional factorial design	663.5 ± 1.43	[135]
*Bacillus natto*	2^5−1^ fractional factorial design, Box–Behnken design and RSM	20.83	[136]
*Bacillus subtilis*	Taguchi experimental design	130.96	[137]
*Bacillus subtilis* ICTF-1	L(18)-orthogonal array method	8814	[138]
*Bacillus sphaericus* MTCC 3672	2*^k^* fractional factorial CCRD, RSM	384	[139]
*Shewanella* sp. IND20	One-factor-at-a-time, e 2^5^ factorial design, RSM, CCD	2751	[140]
*Xylaria curta*	One-variable-at-a-time approach	9.22	[81]
*Pseudoalteromonas* sp. IND11	Two-level full factorial design, RSM, CCD	1573	[141]

**Table 3:** Physiochemical properties of microbial fibrinolytic enzymes.

Fibrinolytic Enzyme	Microbe Associated	Molecular Weight, Optimal pH and Temperature	Functional Moiety	Mechanism of Action	Reference
Streptokinase	*Streptococcus hemolyticus*	47 KDa pH 7.5 37 °C	Single polypeptide chain (414 amino acids) having multiple structural domains (α, β, ϒ)	β domain form SK plasminogen complex resulting in activation of plasminogen	[35]
Staphylokinase	*Staphylococcus aureus*	15.5 KDa pH 8.5 37 °C	Single polypeptide chain (136 amino acids) without disulphide bridge	Higher affinity with traces of plasmin resulting in plasminogen activation	[36]
Serrapeptase	*Serratia marcescens* Strain E 15	45–60 KDa pH 9 40 °C	Metalloprotease containing 3 zinc atoms and one active site	Cleaves peptide bond linkages	[188]
Nattokinase (wild type)	*Bacillus subtilis* YF 38, natto	27.7 KDa pH 8.6	Conserved catalytic triad (Asp^32^, His^64^ and Ser^221^), oxyanion hole (Asn^155^).	Properties resemble plasmin and enhance production of plasmin and clot dissolving agents	[189]
Nattokinase	*Pseudomonas aeruginosa* CMSS	21 KDa 7 pH 25 °C	Similar to wild type nattokinase with two-fold increase in enzymatic activity	Similar to wild type nattokinase	[190]
CK fibrinolytic enzyme	*Bacillus* sp. CK 11-4	28.2 KDa pH 10.5 70 °C	Thermolytic alkaline serine protease (1882 protein atoms, 2 calcium ions and 44 water molecules)	Enhanced production of tissue plasminogen activator	[42]
Fibrinolytic enzyme	*Bacillus* sp. KA38	41 KDa pH 7 40 °C	Metalloprotease	Degrade fibrin or form plasmin from plasminogen	[47]
CFR 15 protease	*Bacillus amyloliquefaciens* MCC2606 (strain CFR 15)	32 KDa pH 10.5 45 °C	Serine protease (catalytic triad: His^57^, Ser^195^, Asp^102^)	Degrade (α polymer, β chain, ϒ-ϓ dimer, α chain) of fibrin	[43]
-	*Bacillus amyloliquefaciens* An6	30 KDa pH 9 60 °C	Serine protease	Degrade fibrin or form plasmin from plasminogen	[191]
Subtilisin DJ-4	*Bacillus* sp. DJ-4	29 KDa pH 10 40 °C	Plasmin-like serine protease	Rapid hydrolysis of α-α, β-β, ϓ chains of fibrin	[37]
Subtilisin QK02	*Bacillus* sp. QK02	28 KDa pH 8.5 55 °C	Subtilisin-family serine protease (Asp 32, His 64 and Ser 221)	Catalytic triad plays an important role in cleaving peptide	[78]
Subtilisin DFE	*Bacillus amyloliquefaciens* DC 4	28 KDa pH 9 48 °C	Serine protease	High specificity towards fibrin and hydrolyse thrombin in vitro	[113]
Fibrinolytic enzyme	*Bacillus tequilensis* CWD-67	22 KDa pH 8 45 °C	Chymotrypsin-like serine metalloprotease containing hydrophobic S1 pocket	Hydrolyse α chain, β chain and finally ϒ-ϓ chain of fibrin	[192]
BacillokinaseII	*Bacillus subtilis* A1	31.4 KDa pH 7 50 °C	Chymotrypsin like serine protease	Digest fibrin as well as act as plasminogen activator	[193]
Fibrinolytic enzyme	*Bacillus* sp. KDO-13	45 KDa pH 7 60 °C	Metalloprotease (Catalytic domain 170 amino acids, hinge region and hemopexin domain of 200 amino acids)	Degrade fibrin or form plasmin from plasminogen	[185]
Fibrinolytic enzyme	*Bacillus* sp. IND 7	32 KDa pH 9	Serine protease	Degrade fibrin or form plasmin from plasminogen	[15]
Bafibrinase	*Bacillus* sp. AS-S20-I	32.3 KDa 7.4 pH 37 °C	Catalytic triad made up of Ser^221^, His^64^ and Asp^32^ and have no intramolecular sulphide bond	Cleave α and β chain of fibrin and fibrinogen	[18]
Subtilisin BK 17	*Bacillus subtilis* BK17	31 KDa	Serine protease	Degrade fibrin or form plasmin from plasminogen	[194]
Fibrinolytic enzyme	*Bacillus subtilis* KCK-7	45 KDa pH 7 60 °C	Serine protease requiring hydroxyl group for activity	Degrade fibrin or form plasmin from plasminogen	[195]
Douchi fibrinolytic enzyme	*Bacillus subtilis* LD 8547	30 KDa	Serine protease	Activate t-PA in vivo	[14]
Fibrinolytic enzyme	*Paenibacillus* sp. IND8	-	-	Degrade fibrin or form plasmin from plasminogen	[196]
SW 1	*Streptomyces* sp. Y405	30KDa pH 8	Serine protease and metalloprotease	Degrade fibrin or form plasmin from plasminogen	[197]
Fibrinolytic enzyme	*Streptomyces rubiginosus* VITPSS1	45 KDa pH 7.2 32 °C	-	Degrade fibrin or form plasmin from plasminogen	[70]
Fibrinolytic enzyme	*Streptomyces* sp. MCMB-379	-	Serine endopeptidase type	Cleaves fibrin fibres by degradation of chains	[198]
β Hemolytic Streptokinase	*Streptococcus equinus*	-	-	Degrade fibrin or form plasmin from plasminogen	[40]
Fibrinolytic enzyme	*Bacillus cereus* SRM-001	28 KDa 7 pH 37 °C	Serine protease	Plasmin catalysed hydrolysis of fibrin	[199]
Fibrinolytic enzyme	*Bacillus cereus* IND 5	47 KDa 8 pH 50 °C	Serine protease	Degrade fibrin or form plasmin from plasminogen	[200]
Fibrinolytic enzyme	*Bacillus pumilus*	20 KDa 50 °C	Serine protease	Degrade α and β chains of fibrinogen but not ϒ chain	[48]
Fibrinolytic enzyme	*Serratia* sp. KG 2-1	8 pH 40 °C	Metalloprotease	Degrade fibrin or form plasmin from plasminogen	[21]
Fibrinolytic enzyme	*Shewanella* sp. IND20	55.5 KDa 8 pH 50 °C	Serine protease	Direct clot lysis and plasminogen activation activity	[140]
Fibrinolytic enzyme	*Cordyceps militaris*	28 KDa 7.2 pH 37 °C	Serine protease	Activate plasminogen to plasmin	[38]
Fibrinolytic enzyme	*Lasiodiplodia pseudotheobromae*	80 KDa	-	Degrade fibrin or form plasmin from plasminogen	[201]
AMMP	*Armillaria mellea*	21 KDa pH 6 33 °C	Chymotrypsin-like metalloprotease	Hydrolyse α-α fibrinogen	[39]
Fibrinolytic enzyme	*Mucor subtilissimus* UCP 1262	20 KDa 40 °C	Chymotrypsin-like serine protease	Activity is similar to plasmin	[106]
Fibrinolytic enzyme	*Cochliobolus lunatus*	pH 6.8 40 °C	-	Degrade fibrin or form plasmin from plasminogen	[202]
Longolytin	*Arthrobotrys longa*	28.6 KDa pH 6–7 37 °C	Serine protease	Degrade fibrin, able to activate plasminogen	[176]
Fibrinolytic protease	*Aspergillus brasiliensis* AUMC 9735	40 KDa pH 8 30 °C	-	Degrade fibrin	[8]
Fibrinolytic protease	*Aspergillus brasiliensis* BCW2	pH 7 45 °C	-	Degrade fibrin	[101]
Fibrinolytic protease	*Aspergillus carbonarius* S-CSR-0007	pH 7 45 °C	-	Degrade fibrin	[31]
Fibrinolytic protease	*Aspergillus fumigatus* CBS 113.26	35 KDa pH 9 37–42 °C	Serine protease	Degrade fibrinogen	[100]
AO-3	*Aspergillus ochraceus* VKM F-4104D	35 KDa pH 9 45 °C	Serine protease	Degrade fibrin	[20]
Terrilytin	*Aspergillus terricola*	27 KDa pH 6.5 53 °C	Serine protease	Degrade fibrin	[19]
Fibrinolytic protease	*Aspergillus ustus* 1	33 KDa pH 6 41 °C	Serine protease	Degrade fibrin	[30]
Fibrinolytic protease	*Aspergillus flavus* O-1	17 KDa	Serine protease	Degrade fibrin	[32]
FP	*Fusarium* sp. BLB	27 KDa pH 9.5 50 °C	Serine protease	Degrade fibrin	[180]
Fu-P	*Fusarium* sp. CPCC480097	28 KDa pH 8.5 45 °C	Chymotrypsin-like serine metalloprotease	Cleaved the α-chain of fibrin (ogen) with high efficiency, and the β-chain and γ-γ (γ-)-chain with lower efficiency	[104]
Fibrinolytic protease	*Neurospora sitophila*	49 KDa pH 7.4 50 °C	Chymotrypsin-like serine protease	Degrade all chains of fibrinogen	[63]
Fibrinolytic protease	*Oidiodendron flavum*	22 KDa pH 8 45 °C	-	Degrade fibrin	[107]
PTEFP	*Paecilomyces tenuipes*	14 KDa pH 5 35 °C	-	Degrade Aα-chain of human fibrinogen but did not hydrolyse Bβ- or γ-chain	[56]
Fibrinolytic protease	*Rhizopus chinensis* 12	16.6 KDa pH 10.5 45 °C	Serine protease and metalloprotease	Degrade fibrin and fibrinogen	[58]
Fibrinolytic protease	*Rhizopus microsporus var. tuberosus*	24.5 KDa pH 7 37 °C	-	Degrade fibrin and activate plasminogen	[62]
Proteinase III	*Sarocladium strictum*	35 KDa pH 10 30 °C	Serine protease	Degrade fibrin and activate plasminogen	[64]
Fibrinolytic protease	*Scedosporium apiospermum*	33 KDa pH 9 37 °C	Subtilisin-like serine protease	Degrade fibrinogen	[110]

**Table 4:** Structural-functional characteristics of microbial fibrinolytic enzymes.

Origin of Enzyme	Structural Prediction	Docking Tools	Substrate/Binding Affinity/Score	Active Sites/ Interacting Residues	References
*Bacillus subtilis* S127e	I-TASSER	-	-	Asp^32^, His^64^ and Ser^221^	[204]
*Bacillus cereus* RSA1	I-TASSER, Swiss-Model, RAPTORX and Phyre2	PATCHDOCK and FIREDOCK	Substrate: Fibrin Affinity: −21.36 kcal/mol	Asp^146^, Ser^164^ and His^132^	[24]
*Bacillus cereus* AB01	I-TASSER	PATCHDOCK	Substrate: Fibrinogen Dock score: 19698	Asp^102^, His^83^ and Ser^195^	[28]
*Bacillus subtilis K2*	SWISS-MODEL	HADDOCK	Substrate: Fibrin Affinity: −19.4 kcal/mol	Asp^19^, His^51^ and Ser^208^	[49]
*Bacillus natto*	MODELLER	AUTODOCK	Substrates: H-D-VLK-pNA and SucAAPF-pNA	Asp^32^, His^64^ and Ser^221^	[205]
*Bacillus subtilis natto*	X-ray diffraction	-	-	-	[206]
*Bacillus subtilis* sp. *natto*	Geno3D2	-	-	-	[207]
*Bacillus* sp.	Protein Data Bank: PDB (4DWW)	HADDOCK	Substrate: Fibrinogen Dock Score: −114.3 ± 4.7	Gly^61^, Ser^63^, Thr^99^, Phe^189^, Leu^209^, Tyr^217^, Asn^218^ and Met^222^	[208]

## Data Availability

All data are available in the main text.
